# Peer Bork (1963–2026): pioneer of computational biology, architect of bioinformatics resources, and visionary microbiome researcher

**DOI:** 10.1093/bioadv/vbag227

**Published:** 2026-09-16

**Authors:** Alex Bateman

**Affiliations:** European Molecular Biology Laboratory, European Bioinformatics Institute (EMBL-EBI), Wellcome Genome Campus, Hinxton, CB10 1SD, United Kingdom

**Figure:**
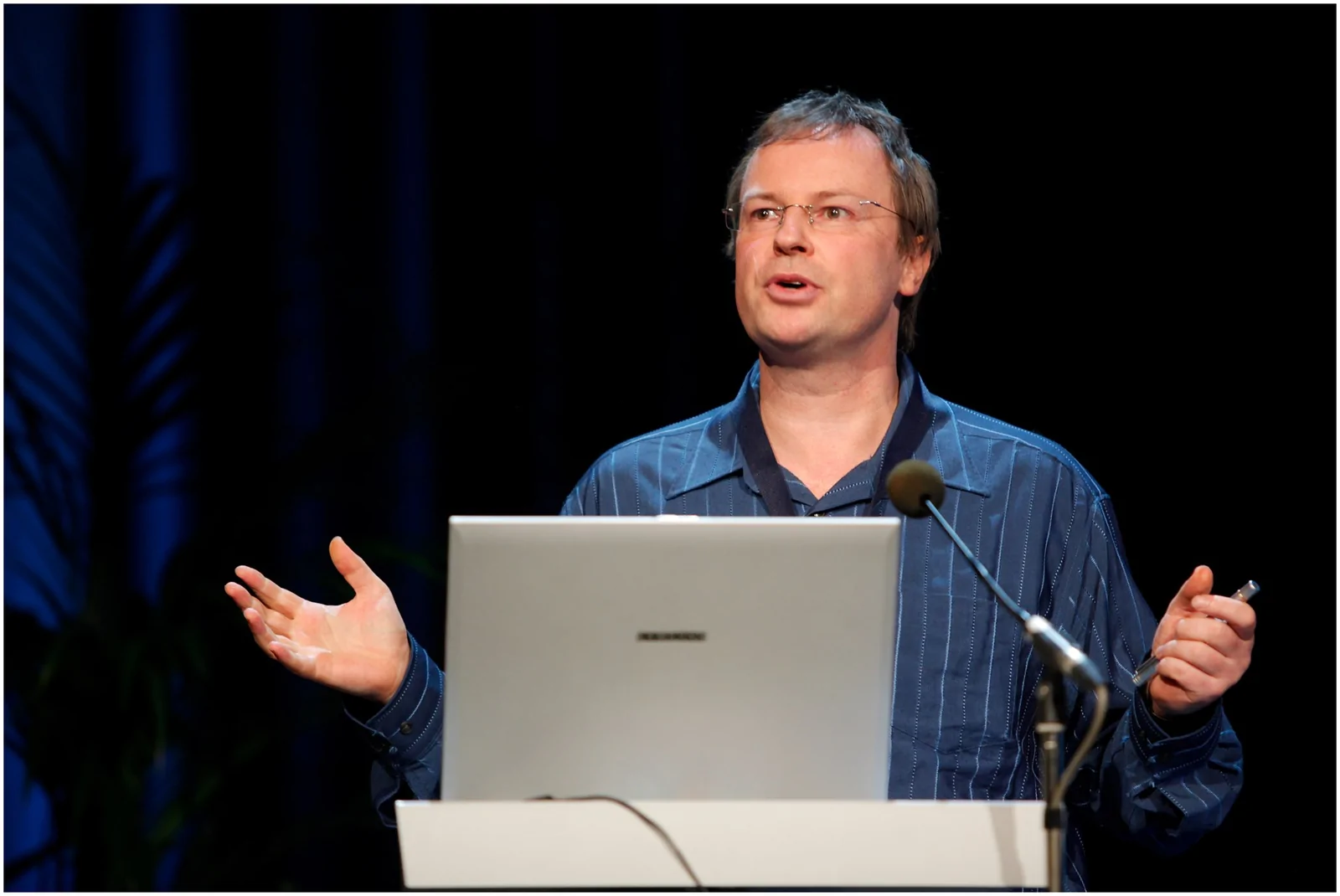
Peer presenting the keynote lecture at the 2010 ECCB conference in Ghent. Photo credit: Rob Stevens. Copyright University of Leuven (2010).

Peer Bork, one of the most influential computational biologists of his generation, died on 16 January 2026 from natural causes (EMBL 2026). He was 62 years old. At the time of his death, he was serving as Interim Director General of the European Molecular Biology Laboratory (EMBL), a role he had held since March 2025 alongside Ewan Birney as Interim Executive Director General. His passing marks the loss of a scientist whose work fundamentally shaped how we understand protein architecture, whose databases and tools became essential infrastructure for biological research worldwide, and whose insights into the human microbiome opened entirely new fields of investigation.

## From East Berlin to bioinformatics

Peer was born on 4 May 1963 and spent his formative years in East Berlin during the Cold War era (Fogg *et al.* 2021). Mathematics captured his imagination early; he excelled in national-level competitions and was admitted to one of East Germany’s selective schools for mathematically gifted students. It was there, in the early 1980s, that he first encountered programming, working on an enormous Soviet-built machine that filled much of a room.

Yet Peer’s intellectual appetite extended well beyond mathematics. He read widely in the natural sciences, developing a particular curiosity about the molecular machinery underlying life. Later, reflecting on why he chose a scientific career, he offered a characteristically candid explanation: “I was always a curious person who used analytical thinking, and having grown up in East Germany, science was an area where I hoped that I could stick to facts with a limited impact from political propaganda” (Fogg *et al.* 2021).

At the University of Leipzig, where he studied biochemistry, Peer discovered the power of combining computation with biology. His diploma project in 1987 examined how enzyme cofactor-binding domains had evolved, work that required him to gather sequences from the nascent protein databases of the era, develop his own pattern-matching algorithms, and piece together evolutionary relationships from fragmentary data. This apprenticeship in sequence analysis would prove foundational to everything that followed.

## A remarkable PhD supervisor

Peer began his doctoral research in 1988 under Jens Reich, a theoretical biophysicist whose life exemplified the tensions of that historical moment. Reich was simultaneously running a research group and serving as a leading figure in East Germany’s pro-democracy opposition, activities that placed him under persistent secret police surveillance and a real threat of imprisonment. After reunification, Reich entered politics, eventually standing as a candidate for the German presidency.

Despite the extraordinary pressures on him, Reich proved a formative mentor. Peer later recalled: “He never made a fuss about his political standing, and at work he encouraged and helped me without micromanagement. He treated everybody equally, was a knowledgeable and engaging supervisor and supported me, even during unification, when everyone at my institute lost their jobs and had to re-apply for the few new positions” (Fogg *et al.* 2021). Working in a rapidly expanding field where computational insights came quickly, Peer completed his doctorate in biochemistry in just 18 months, graduating in 1990.

It was during this period that Chris Sander, then at EMBL, visited Reich’s group in East Berlin, before the Wall came down, and recognized Peer’s exceptional talent. Sander invited him to EMBL, first for a short visit and then as a postdoc. They would go on to publish some 20 papers together over the following years.

## The modular LEGO principle: pioneering domain discovery

The late 1980s and early 1990s represented a unique window in computational biology. Sequence databases were growing rapidly but remained small enough that a single researcher with the right computational tools could systematically mine them for biological insights. Peer seized this opportunity. As he later recalled: “It was not uncommon to get results sufficient for a good scientific paper within a week or so, and as a byproduct, one could learn a lot about molecular biology while reading the papers around the published protein sequences” (Fogg *et al.* 2021).

What began as studies of enzymatic domains evolved into a broader program to understand protein modularity. Peer recognized that proteins are assembled from a finite vocabulary of conserved structural units, “the modular LEGO principle of building blocks for protein function,” as he termed it (Fogg *et al.* 2021). He began cataloging newly identified domains in his own database, laying the groundwork for what would become SMART.

The harvest from this period was remarkable. Peer and his colleagues identified or systematically characterized numerous protein domains that are now textbook knowledge: the WW domain, a compact protein-interaction module defined by two signature tryptophan residues (Bork and Sudol 1994); the SEA module found in heavily glycosylated cell-surface proteins (Bork and Patthy 1995); HEAT repeats, whose discovery in the Huntingtin protein provided early molecular insight into Huntington’s disease (Andrade and Bork 1995); and the BRCT domain, present in BRCA1 and other proteins involved in responding to DNA damage (Bork *et al.* 1997). These discoveries established sequence-based approaches as powerful tools for predicting protein function and illuminating cell biology.

## Three decades at EMBL

Peer joined EMBL Heidelberg in 1991 and would remain affiliated with the organization for the rest of his life. He completed his Habilitation in theoretical biophysics at Humboldt University Berlin in 1995, the same year he became an EMBL Group Leader. In 2001, he was appointed Head of the Structural and Computational Biology Unit, a position he held for two decades while also serving from 2011 as Strategic Head of Bioinformatics across EMBL. He became Director of EMBL Heidelberg (Scientific Activities) in 2020, and in March 2025 was appointed Interim Director General.

Throughout his career, Peer also held honorary professorships at the Universities of Heidelberg and Würzburg and at Fudan University in Shanghai, and received honorary doctorates from Würzburg (2014), Utrecht (2017), and Copenhagen (2024).

## Building the infrastructure of computational biology

If Peer’s domain discoveries established his scientific reputation, his databases and tools cemented his influence on the daily practice of biological research. SMART, launched in 1997 and still actively maintained as it approaches its 30th anniversary, enables researchers worldwide to analyze the domain architecture of proteins (Letunic and Bork 2025). STRING revolutionized the prediction of protein functional associations by exploiting genomic context, the observation that genes encoding interacting proteins tend to be neighbors on chromosomes across multiple species (Szklarczyk *et al.* 2023). The eggNOG database now provides orthology assignments across >12 500 organisms (Hernández-Plaza *et al.* 2023); proGenomes offers curated prokaryotic reference genomes (Offner *et al.* 2025); and iTOL has become a standard tool for visualizing and annotating phylogenetic trees (Letunic and Bork 2021).

Crucially, Peer understood that databases require sustained community effort to remain useful. Many of these resources continue to be developed by international teams, frequently led by scientists who trained in his group. This ecosystem of maintained, interoperable resources represents one of his most enduring contributions to the field.

## Transforming microbiome science

From the mid-2000s, Peer increasingly directed his computational expertise toward understanding microbial communities. “I moved research subfields a few times but got stuck on microbiomes for more than 15 years,” he later reflected, “mostly focusing on the human gut microbiome” (Fogg *et al.* 2021).

His most influential contribution to this field came in 2011, when his team reported in Nature that human gut microbiomes cluster into distinct community types, enterotypes, characterized by different dominant bacterial genera (Arumugam *et al.* 2011). Now cited nearly 10 000 times, the paper reshaped thinking about inter-individual variation in the gut microbiome. Yet Peer himself remained characteristically humble about the finding: “I found our discovery of enterotypes surprising, as we could not really explain it, and I think, despite tons of hypotheses, still nobody can really explain this observation” (Fogg *et al.* 2021).

Peer’s microbiome work extended far beyond the human gut. He was a central figure in the Tara Oceans expedition, contributing computational analyses to landmark 2015 papers characterizing the global ocean microbiome (Sunagawa *et al.* 2015). He co-led the MetaHIT consortium, which cataloged millions of microbial genes from human samples (Qin *et al.* 2010). And he maintained an expansive vision for where the field might lead: “I believe that the progress of the gut microbiome research towards improving human health can be paralleled by global microbiomics towards planetary health… It seems possible to get a census and understanding of molecular and cellular functions and their evolution for our entire planet” (Fogg *et al.* 2021).

## Recognition and honors

Peer’s contributions earned wide recognition. In 2021 alone, he received both the Novozymes Prize (DKK 5 million) from the Novo Nordisk Foundation and the ISCB Accomplishments by a Senior Scientist Award, the International Society for Computational Biology’s highest honor (Fogg *et al.* 2021). Earlier accolades included the 2009 Microsoft Award from the Royal Society and Académie des Sciences and the 2008 Nature Award for Creative Mentoring.

He was elected to the German National Academy of Sciences Leopoldina in 2014, became an EMBO member in 2000, and was a member of Academia Europaea. He held two ERC Advanced Investigator Grants.

## Mentor and entrepreneur

The Bork group served as a training ground for computational biologists worldwide; >50 of Peer’s former trainees now lead their own research groups or hold professorships. His mentoring philosophy, informed by his own experience with Jens Reich, emphasized giving students intellectual freedom while helping them situate their findings within broader scientific contexts. He was a prolific teacher through EMBO courses and contributed to the scientific community as Senior Editor of Molecular Systems Biology and through editorial service for Science and Cell.

Peer also bridged academia and industry, co-founding several biotechnology companies: LION Bioscience AG (1997), Cellzome AG (1999; later acquired by GlaxoSmithKline), Anadys Pharmaceuticals (2000), and biobyte solutions GmbH (2001).

## A data-driven visionary

Peer’s career was marked by a willingness to venture into new territory. He gravitated toward emerging fields where established methods had yet to be developed, letting the data guide his investigations rather than testing preconceived hypotheses. This approach allowed him to move fluidly between protein structure prediction, genome annotation, network biology, and microbiome research as each field matured and new frontiers beckoned.

The scale of his output was extraordinary: >600 publications, over 80 papers in Nature, Science, or Cell, a citation count exceeding 460 000, and an h-index above 280. But statistics capture only part of his impact. The databases he built and the community he nurtured have enabled discoveries by countless researchers who may never have encountered his papers directly but whose work rests on the infrastructure he created.

## Remembering Peer

Those who knew Peer invariably comment on his extraordinary energy and intellectual intensity. Samuel Chaffron, who began as an undergraduate intern in Peer’s lab 20 years ago, speaks for many whose careers were shaped by him: “This was a key moment in my life that shaped my scientific career forever. Peer was always forward-looking, inspiring, and communicating a scientific energy that was contagious.”

Sarah Teichmann, who had Peer as her external PhD examiner in 1999, remembers “three intense hours debating genome and protein analysis. Peer was full of energy and intensity, which continued to be a hallmark of his delightful personality right over the following decades.” Martin Beck captures another dimension of Peer’s scientific persona: “His scientific creativity was so extraordinary that one often wondered whether he possessed some intellectual advantage over us, ordinary scientists. He could edit a dull scientific paper so that it suddenly became an exciting read. He could collapse an elaborate proposal like a house of cards with a single question.”

But Peer was far more than his science. He had wide-ranging cultural interests: Shoba Ranganathan recalls his love of poetry from around the world and his enthusiasm for Michael Ondaatje’s “The Cinnamon Peeler.” He knew how to have fun: Mihai Pop fondly remembers sneaking out of an ISMB conference with Peer to watch Germany play in a World Cup final at a bar near the Boston Convention Center. And Martin Beck notes that “Peer was the grand master of Liar’s Dice. He certainly knew how to throw a party. And he had style.”

Madan Babu highlights what made Peer’s scientific thinking distinctive: “His ability to connect the smallest observation to the largest question, and to do so with humility, rigor, and a distinct joy for discovery.” Ruedi Aebersold describes him as “a fountain of ideas that he generously shared” and “a warm and emphatic human being.” Oli Stegle remembers him as “visionary, kind, and wonderfully unconventional.”

Christine Orengo recalls meeting him >30 years ago, on a bus to an EMBO conference: “He was a PhD student and I was a postdoc. I was amazed by his energy, focus, and sheer passion for his work. He seemed scientifically mature way beyond his years.” She later collaborated with him: “It was one of the fastest and most fun collaborations I ever had. He had incredible drive. He was a pioneer and fearlessly went into new biological territory and built all the necessary tools to make robust new discoveries.”

Peer Bork’s influence on computational biology, through the domains he discovered, the databases he built, the fields he helped create, and the scientists he trained, will endure for generations. Further tributes can be found in his online condolence book (https://peerbork.muchloved.com).
